# Editorial: CD4 T Follicular Helper Cells in HIV

**DOI:** 10.3389/fimmu.2017.01894

**Published:** 2017-12-22

**Authors:** Smita S. Iyer

**Affiliations:** ^1^Department of Pathology, Microbiology, and Immunology, Center for Comparative Medicine, The California National Primate Research Center, University of California at Davis, Davis, CA, United States

**Keywords:** antibody, target cells, reservoirs, vaccine, prevention

**Editorial on the Research Topic**

**CD4 T Follicular Helper Cells in HIV**

CD4 T follicular helper cells are vital for induction of long-lived humoral immunity; this specialized function makes them highly desirable immunological targets for designing an effective HIV vaccine. Intriguingly, T_FH_ cells could also hold clues to a functional cure for HIV; as reservoirs of latent HIV during antiretroviral therapy (ART), T_FH_ cells facilitate HIV persistence. This special topic “CD4 T_FH_ cells in HIV” synthesizes reviews from experts in the field to explore, discuss, and share recent discoveries on the intricate relationship between T_FH_ cells and HIV. This special topic also seeks to offer perspectives on areas needing further inquiry in this rapidly evolving field.

Despite the success of ART, achieving a functional cure for HIV, i.e., sustained viral suppression after ART interruption, remains a challenge due to persistence of viral sanctuaries under ART. As a result, there is a critical need to understand the reservoir to devise effective strategies to purge the latent virus and eliminate life-long dependence on ART. In this special topic, experts in the HIV field synthesize and evaluate recent discoveries in humans and non-human primate model systems to understand the complex dynamics of T_FH_ cells during HIV infection. Leong et al. delineate potential cell intrinsic and extrinsic factors driving viral replication within T_FH_ cells during HIV infection. These include lack of cellular host restriction factors within T_FH_ cells, exclusion of cytolytic CD8 T cells from B cell follicles within germinal centers (GCs), and trapped virus on GC follicular dendritic cells; a constellation of factors that create a perfect storm to promote unfettered viral replication within GC T_FH_ cells.

Surprisingly, despite being hotbeds for viral replication, T_FH_ cells are not depleted as dramatically as other CD4 T cell subsets. Zaunders et al. report a clear increase in proportion of T_FH_ cells compared to other CD4 T cell subsets in serial fine-needle aspirates from lymph nodes of SIV-infected macaques. Miles et al. reveal that an increase in interleukin 6 and interferon gamma and a corresponding reduction in IL-2 could promote T_FH_ proliferation. Hong et al. and Wang et al. argue that a relative increase in T_FH_ cell proportions occurs in the face of net decrease in T_FH_ cells numbers. They attribute the decrease in T_FH_ numbers to increased expression of programmed death-ligand 1 on dendritic cells following infection. Hong et al. also state that an increase in transforming growth factor-b-mediated collagen deposition and fibrosis and loss of fibroblastic reticular cells drives disruption of the lymphoid architecture impairing T_FH_ cell numbers and function. Miles et al. state that an increase in T follicular regulatory cells impairs T_FH_ function in HIV infection. While these observations may seem at odds with each other, given the complex immunopathology of HIV infection and progression to AIDS, it is highly likely that T_FH_ cell numbers and function vary widely over the course of infection and between infected animals and humans. Ultimately, B cell responses are impaired with aberrant B cell activation and hypogammaglobulinemia in the face of poor HIV-specific antibody responses. Chiodi et al. raise the possibility that IL-7, a cytokine whose level is elevated during HIV-1 infection, may have a role in increased expression of B cell costimulatory molecules on T_FH_ cells leading to abnormal B cell differentiation and apoptosis.

However, antibody responses are not compromised in all HIV-infected individuals. In some infected individuals, broadly neutralizing antibodies that have acquired extensive hypermutation and even deletion/insertion mutations appear several years after initial infection. Zaunders et al. contend that not all immune responses are impotent but that effective immune responses drive virus evolution resulting in Envelope divergence and diversification. The generation of neoantigens in the infected host could recruit naive CD4 T cells, which upon activation drive viral replication leading to eventual depletion of CD4 T cells.

In their review, Graff-Dubois et al. assert that T_FH_ cell frequency varies widely according to (i) the stage of HIV/SIV infection, (ii) the severity of the disease, and (iii) the ability to develop broadly neutralizing antibodies highlighting that a blanket increase or decrease in T_FH_ cells is not a unifying characteristic of all HIV/SIV infections. Given the intricate link between virus and lymph node cells, blocking HIV replication in lymphoid tissues might be a prerequisite for induction of effective T_FH_ responses and anti-HIV antibodies and could have the benefit of dramatically decreasing seeding of the latent reservoir. While the lymph node has received a great deal of attention, other compartments are also emerging as critical parameters deserving of attention.

Thornhill et al. review what is known about peripheral(p) T_FH_ cells, which are now emerging as valuable surrogates for GC T_FH_ cells and indicate that the preservation of pT_FH_ cells during HIV infection by early ART initiation is associated with better viral suppression and lack of B cell dysfunction. Moukambi et al. highlight the importance of T_FH_ cells in the spleen, the primary organ for B cell activation and differentiation. Their recent observations indicate early and profound loss of splenic T_FH_ cells. There is also emerging interest in studying T_FH_ cells in mucosal compartments—this understanding of the T_FH_ cell response in distinct tissue compartments with differences in viral dynamics will undoubtedly shed further insights in the relationship of CD4 T_FH_ cells and HIV. Thus, there remains a great deal to learn about T_FH_ cells, and as with HIV, these cells are likely to attract the attention of immunologists and virologists alike.

## Author Contributions

The author confirms being a contributor of this work and approved it for publication.

## Conflict of Interest Statement

The author declares that the research was conducted in the absence of any commercial or financial relationships that could be construed as a potential conflict of interest.

